# Inflammation-Related Parameters in Lung Cancer Patients Followed in the Intensive Care Unit

**DOI:** 10.3390/healthcare14010039

**Published:** 2025-12-23

**Authors:** Burcu Tunay, Omer Fatih Olmez, Ahmet Bilici, Ayberk Bayramgil, Gunes Dorukhan Cavusoglu, Huseyin Oz

**Affiliations:** 1 Department of Anesthesiology and Reanimation, Istanbul Medipol University, Istanbul 34000, Turkey; hoz@medipol.edu.tr; 2 Department of Medical Oncology, Faculty of Medicine, Medipol University, Istanbul 34000, Turkey; ofolmez@gmail.com (O.F.O.); ahmetknower@yahoo.com (A.B.); 3 Department of Internal Medicine, Istanbul Medipol University, Istanbul 34000, Turkey; ayberkbayramgil@gmail.com (A.B.); gunescavusoglu@gmail.com (G.D.C.)

**Keywords:** lung cancer, mortality, APACHE II, prognostic nutritional index, predictive value of test

## Abstract

**Objectives:** Lung cancer remains as the most common cause of cancer-related death. The possible relationships between inflammatory markers and lung cancer prognosis have yet to be clarified. In this study, we aimed to assess and compare various inflammatory markers and prognostic tests for their role in predicting mortality in patients with lung cancer who were admitted to the intensive care unit. **Methods:** A total of 229 patients diagnosed with small cell or non-small cell lung cancer who attended follow-up after treatment were included. The predictive performance of neutrophil-to-lymphocyte ratio (NLR), platelet-to-lymphocyte ratio (PLR), lymphocyte-to-monocyte ratio (LMR), systemic immune-inflammation index (SII), modified Glasgow prognostic score (mGPS), Prognostic nutritional index (PNI), APACHE II score, and MPM II-Admission (Mortality Probability Models II-0) were assessed in terms of mortality status. We also performed multivariable logistic regression to determine whether any of these parameters were independently associated with mortality. **Results:** We included 229 patients into our study; the mean age was 66.17 ± 11.89 years. Among these, 135 (58.95%) patients died and 94 (41.05%) patients were discharged. When we evaluated the performance of the prognostic scores in predicting mortality, we found mGPS, MPM II-Admission, and APACHE II scores had the highest sensitivity, and MPM II-Admission, PNI, and APACHE II scores had the highest specificity. Multivariable regression revealed that PNI was the only inflammation-related parameter that was independently associated with mortality. **Conclusions:** PNI, APACHE-II, and MPM II-Admission may be used as easily accessible tests for mortality estimation in lung cancer patients admitted to the ICU.

## 1. Introduction

Lung cancer remains the most important cause of cancer-related death and the second most common cancer diagnosed in both men and women, with approximately 1.8 million people affected annually throughout the world [1,2]. History of cancer and cancer-related problems constitute approximately 17% of the reasons for admission to intensive care units (ICUs) [3]. In early studies, mortality ranged from 75% to 91% in such patients; however, mortality rates have demonstrated a decrease over time [4,5]. As in all types of cancer, it is difficult to predict the consequences of disease and mortality in lung cancer. Therefore, there is a need for biomarkers that can predict the outcome of the disease and thus help identify the patients most likely to benefit from treatments and to determine management [2].

Extensive research has been conducted to find the best prognostic and predictive laboratory markers for lung cancer and many other types of cancer [6,7,8]. This is due to the fact that inflammation is a well-established triggering factor for the formation and development of cancer. For instance, systemic inflammatory response markers such as plasma C-reactive protein (CRP), hypoalbuminemia, and absolute white cell count (WBC) are associated with cancer development and progression [8]. Furthermore, several markers which are calculable from routine hemogram parameters, including neutrophil-to-lymphocyte ratio (NLR), platelet-to-lymphocyte ratio (PLR), lymphocyte-to-monocyte ratio (LMR), and the systemic immune-inflammation index (SII), are known to be strong prognostic markers associated with worse overall survival (OS) in various tumor types, including lung cancer in the pre-immunotherapy period [2,6,7]. On the other hand, there are also several scoring systems that are utilized to assess prognosis in various conditions including squamous and non-squamous lung cancer, such as the Glasgow prognostic score (GPS), Acute Physiology and Chronic Health Evaluation (APACHE), Prognostic nutritional index (PNI), and Mortality Probability Models (MPM) [2,4,9,10,11]. However, despite many studies concerning inflammation scores and other tests in the literature, it is still unclear which inflammation score best predicts prognosis in patients with lung cancer. In addition, the biological mechanisms underlying the prognostic value of inflammation scores have not been explained yet [12].

In this study, we aimed to evaluate the value of various inflammatory markers and tests in terms of their predictive capability for mortality in patients with lung cancer who were admitted to the ICU for any reason, and to identify the strongest predictor.

## 2. Material and Methods

### 2.1. Study Design

This retrospective cohort study was conducted between 2014 and 2020 at the Medical Oncology Department and Intensive Care Unit, Medipol University Faculty of Medicine, Istanbul, Turkey. Approval was obtained from the ethics committee of Medipol University for the study (date: 18 November 2021, decision no: E-10840098-772.02-5923). All samples and information were recorded anonymously.

The study timeframe of 2014–2020 was selected to ensure consistency of electronic medical records, as our institution transitioned to a unified digital documentation system in early 2014. This period also provided sufficient sample size for robust statistical analysis given the relatively small ICU lung cancer population. Moreover, ICU management protocols for ventilatory support, vasopressor use, and oncology co-management remained stable during these years, minimizing temporal bias.

### 2.2. Patients and Data Collection

This study includes 229 patients diagnosed with small cell and non-small cell lung cancer who were followed up with and treated in the Medical Oncology Department of Medipol University Faculty of Medicine, Istanbul, Turkey, between 2014 and 2020 and required admission to the ICU at any stage of their treatment. All patients included in the study were older than 18 years of age (age range: 22–92 years). We excluded patients younger than 18 years of age, those with concomitant infections such as human immunodeficiency virus or hepatitis, patients who used systemic steroids or anti-inflammatory or immunosuppressive medicine, subjects with autoimmune disorders or a history of other malignancies, and individuals with missing information regarding the parameters evaluated in the study. Patients were grouped with respect to mortality status.

Missing data were handled using complete-case analysis for <5% missingness and multiple imputation (MICE) for 5–20% missingness; variables exceeding 20% missingness were excluded from multivariable models.

We recorded patients’ age, sex, lung cancer type, comorbidity status, chemotherapy status, malignancy status, tumor stage, reason of ICU admission, length of stay in the ICU, mechanical ventilation (MV) and invasive MV (IMV) status, laboratory parameters including hemoglobin, platelet, neutrophil, lymphocyte, eosinophil, monocyte, albumin, WBC, CRP, lactate dehydrogenase (LDH), and inflammatory and prognostic scores including NLR, PLR, LMR, SII, Modified Glasgow prognostic score (mGPS), PNI, APACHE-II score, and MPM II-Admission (Mortality Probability Models II-0). If not stated otherwise, all calculations were made according to baseline findings and laboratory values obtained from blood drawn at ICU admission.

Briefly, NLR was defined as the total number of neutrophils divided by the total number of lymphocytes, PLR was defined as the total number of platelets divided by the total number of lymphocytes, and LMR was defined as the total number of lymphocytes divided by the total number of monocytes. SII was calculated with the following formula: SII = peripheral platelet counts × neutrophil counts /lymphocyte counts [13]. The APACHE-II score is composed of 12 acute physiological parameters (acute physiology score), patient age, chronic diseases, and surgical procedures, and was calculated as described previously [14] after the patient completed the first 24 h in the ICU. The mGPS was calculated with respect to thresholds for CRP and albumin values: A CRP value of ≤8 mg/L and albumin value of ≥35 g/L indicated an overall score of 0; if one was abnormal the score was 1, and if both were abnormal the score was 2 [15]. Preoperative PNI was calculated using the following formula: 10 × serum albumin levels (g/dL + 0.005 × total lymphocyte count (per mm^3^ [16]. MPM II-Admission was determined according to 15 variables (age, coma or deep stupor, heart rate ≥ 50 beats/min, systolic blood pressure ≤ 90 mm Hg, chronic renal insufficiency, cirrhosis, metastatic neoplasm, acute renal failure, cardiac dysrhythmia, cerebrovascular incident, gastrointestinal bleeding, intracranial mass effect, cardiopulmonary resuscitation prior to admission, mechanical ventilation, and medical or unscheduled surgery admission) [17].

### 2.3. Statistical Analysis

All statistical analyses conformed to a significance threshold of *p* < 0.05 and were performed on SPSS ver. 25.0 (SPSS Inc., Chicago, IL, USA). Normality of distribution was assessed via histogram and Q-Q plots. Data are described with mean ± standard deviation or median (1st quartile–3rd quartile) for continuous variables (respecting parametric assumptions), and with frequency (percentage) values for categorical variables. Normally distributed variables were analyzed with an independent samples t test. Non-normally distributed variables were analyzed with the Mann–Whitney U test. Categorical variables were analyzed with chi-square tests or Fisher’s exact tests. Prediction performance of the variables was evaluated by using Receiver Operating Characteristic (ROC) curve analysis, and the following values were calculated: area under ROC curve (AUC) sensitivity, specificity, positive predictive value (PPV), and negative predictive value (NPV). Multiple logistic regression analysis (forward conditional method) was performed to determine the prognostic factors best associated with mortality. Mechanical ventilation and vasopressor initiation were not included in the multivariable model because they were highly collinear with APACHE II and MPM II-Admission, and their inclusion resulted in model overadjustment. 

Model validity was assessed using the Hosmer–Lemeshow goodness-of-fit test, and multicollinearity was evaluated using variance inflation factors (VIFs); all VIF values were <2.0, indicating no significant collinearity. Optimal ROC cut-off points were determined using Youden’s J statistic for all variables.

Advanced metrics such as NRI or decision-curve analysis were not feasible due to retrospective design and sample size. Missing data were handled according to predefined thresholds. Variables with <5% missingness were analyzed using complete-case (listwise) methods. For variables with 5–20% missingness, multiple imputation with chained equations (MICE) was performed, as missingness was judged to be at random. Variables with >20% missingness were excluded from multivariable models to avoid imputation-driven bias. Separate analysis of NSCLC and SCLC was not performed due to the small number of SCLC patients, which did not allow for reliable statistical subgroup comparisons. Survival was selected as the primary endpoint because it is the most objective and clinically meaningful outcome in critically ill cancer patients, whereas other potential outcomes (organ support, vasopressor use, ventilatory parameters, and length of stay) were highly collinear with APACHE II and MPM II scores and inconsistently available in this retrospective dataset.

## 3. Results

The study included 229 patients (163 males and 66 females); mean age was 66.17 ± 11.89 (range 22–92) years. Among these, 135 (58.95%) patients died (exitus group) and 94 (41.05%) were discharged from the hospital. There were no significant differences between the exitus and discharged groups in terms of age, sex, diagnosis (small cell lung cancer and non-small cell lung cancer), and comorbidities (*p* > 0.05, for all).

The frequency of recurrence/progression was significantly higher in the exitus group than in the discharged group (*p* < 0.001). Respiratory problems and sepsis frequencies were significantly higher in the exitus group than in the discharged group and postoperative admission percentage was significantly higher in the discharged group than in the exitus group (*p* < 0.001). MV (*p* < 0.001) and IMV (*p* < 0.001) were used significantly more frequently in the exitus group compared to the discharged group. We found no significant differences between the two groups in terms of cancer stage and length of stay in the ICU.

Hemoglobin (*p* < 0.001), platelet (*p* = 0.007), lymphocyte (*p* = 0.017), eosinophil (*p* = 0.002), and albumin (*p* = 0.004) levels were significantly higher in the discharged group than in the exitus group. CRP (*p* = 0.001) and LDH (*p* = 0.009) were significantly higher in the exitus group than in the discharged group. We found no significant differences between the groups in terms of WBC, neutrophil, and monocyte counts (*p* > 0.05 for all). NLR (*p* = 0.016), mGPS (*p* < 0.001), APACHE II score (*p* < 0.001), and MPM II-Admission score (*p* < 0.001) were significantly higher in the exitus group than in the discharged group. PNI (*p* = 0.004) was significantly higher in the discharged group than in the exitus group. We found no significant differences between groups in terms of PLR, LMR, and SII (*p* > 0.05 for all) (Table 1).

**Figure 1:**
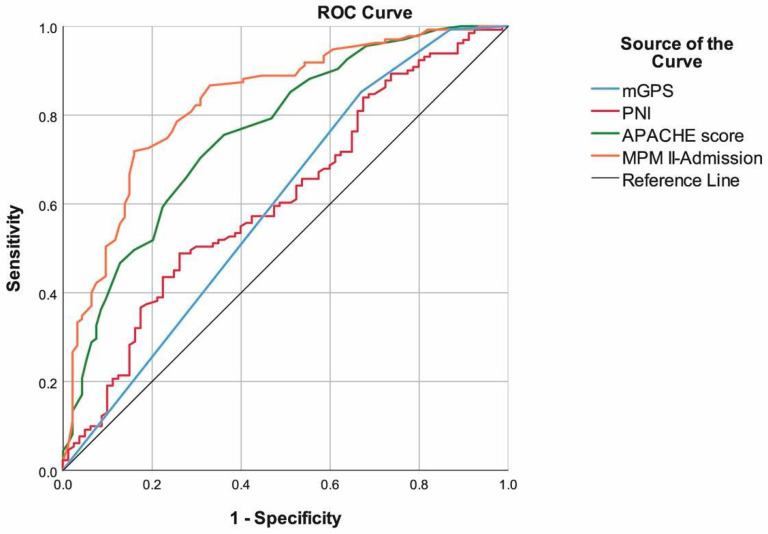
ROC curve of the NLR, PLR, LMR, and SII to predict mortality.

**Figure 2:**
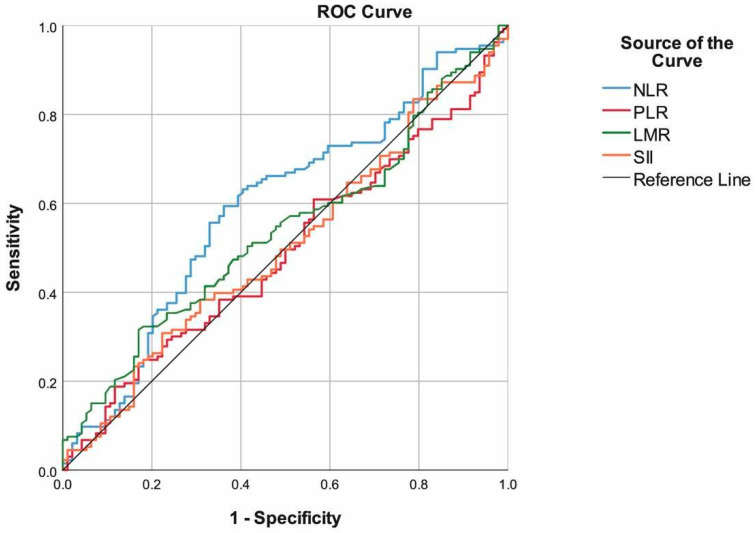
ROC curve of the mGPS, PNI, APACHE score, MPM II-Admission to predict mortality. Figure legend includes AUC values with 95% confidence intervals.

When we evaluated the performance of the prognostic scores in predicting mortality, we found mGPS (85.19%), MPM II-Admission (71.85%), and APACHE scores (70.37%) had the highest sensitivity. MPM II-Admission (84.04%), PNI (73.75%), and APACHE scores (69.15%) had the highest specificity. Additionally, MPM II-Admission (AUC: 0.829, 95% CI: 0.775–0.884), APACHE score (AUC: 0.762, 95% CI: 0.699–0.824), and PNI (AUC: 0.611, 95% CI: 0.53–0.689) had the highest area under the ROC curve. The prediction performances of the PLR, LMR, and SII were found to be non-significant (Table 2, Figure 1 and Figure 2). ROC analyses were further detailed to highlight the comparative discriminatory performance of inflammation-based markers versus severity-based scores. Ninety-five percent confidence intervals for AUC values have been added to the ROC figures to enhance interpretability. Figure legends were revised for clarity, and AUC values with their corresponding 95% confidence intervals are now displayed directly on each ROC curve.

We performed multiple logistic regression analysis to determine prognostic factors that were best associated with mortality. We found presence of recurrence/progression, low PNI (<31.1), high APACHE score (≥20), and high MPM II-Admission score (≥59) to be poor prognostic factors, while being admitted to the ICU in the postoperative period was determined to be a good prognostic factor. Patients with recurrence/progression had a 6.553-fold higher risk of death than those without (OR: 6.553, 95% CI: 2.446–17.553, *p* < 0.001). Postoperative patients had a 31.250-fold lower risk of death than those admitted for other reasons (OR: 0.032, 95% CI: 0.004–0.271, *p* = 0.002). Patients with low PNI (<31.1) had a 2.559-fold greater risk of death than those with higher PNI (OR: 2.559, 95% CI: 1.014–6.455, *p* = 0.047). Patients with a high APACHE II score (≥20) had a 2.860-fold greater risk of death than other patients (OR: 2.860, 95% CI: 1.165–7.024, *p* = 0.022). Patients with a high MPM II-Admission score (≥59) had a 29.527-fold greater risk of death than those with lower scores (OR: 29.257, 95% CI: 8.635–99.128, *p* < 0.001). Other variables included in the model, age (*p* = 0.753), sex (*p* = 0.895), chemotherapy administration (*p* = 0.724), respiratory problems (*p* = 0.969), sepsis (*p* = 0.401), NLR (*p* = 0.278), PLR (*p* = 0.347), LMR (*p* = 0.665), SII (*p* = 0.560), and mGPS (*p* = 0.408), were found to be non-significant (Table 3). 

Additional adjustment using available chemotherapy exposure and comorbidity index did not materially change the association between PNI and mortality. 

## 4. Discussion

The relationship between inflammation and cancer has been the subject of numerous studies after the determination of associations many years ago [18,19,20]. The role of inflammation in the etiopathogenesis of lung cancer has been defined, and many studies have confirmed the prognostic value of inflammation in both local and advanced lung cancer, but data is limited regarding overall analyses for various markers or scores [21]. For this purpose, we recorded relevant markers in lung cancer patients who had been admitted to the ICU and compared results in patients who had been discharged or died. Results revealed low to moderate levels of discrimination ability in various scores, but more importantly, multivariable analysis showed that recurrence/progression, low PNI, high APACHE II, and high MPM II-Admission were indicative of poor prognosis in patients who were admitted to the ICU and had lung cancer. Of note, we also found that mGPS, MPM II-Admission, and APACHE scores had the highest sensitivity to identify patients that had died during their ICU stay, whereas MPM II-Admission, PNI, and APACHE scores had the highest specificity for the same purpose. Lastly, although it can be seen as an expected result, postoperative admission was identified as a good prognostic factor.

The power of various inflammation scores to predict the prognosis of many types of cancer has been and continues to be an important topic of interest to many researchers. PNI is an indicator of systemic immune nutritional status and its levels are associated with the interplay between inflammation, immunological status, and nutrition. It is a well-known prognostic biomarker in lung cancer patients [10,16]. Kitahara et al. found that preoperative PNI level was strongly associated with postoperative outcomes in lung cancer patients. They stated that their low-PNI group had shorter recurrence-free survival and overall survival compared to patients with high PNI, and they argued that PNI was an independent factor in determining lung cancer prognosis [16]. The results of another study demonstrated that the PNI score is useful in evaluating the overall survival of lung cancer patients, and it was emphasized in this study that PNI was valuable as a cost-effective prognostic marker; therefore, it should be included in routine clinical practice [22]. Furthermore, another study put forth that low PNI was an independent predictor of worse prognosis for patients with lung cancer [10]. Looking at the results of the present study, we also found a significant association between low PNI and mortality in multivariable analysis, consistent with the results of previous studies. This is in addition to the fact that PNI was one of the tests that was found to have significant prognostic power, as determined by ROC analyses showing PNI to be the second-best test for specificity, and the third-best test with respect to PPV and AUC.

The APACHE II is a well-established disease classification system that is widely used for risk stratification of critically ill patients in the ICU [14]. In a study employing multivariable analyses, it was shown that APACHE II, female sex, need for intubation, and need for IMV were independently associated with higher ICU mortality and it was emphasized that these predictors should be taken into account for the survival expectations of these patients in the ICU [4]. In another study conducted by Kuo et al., it was shown that the presence of active newly diagnosed cancer, active recurrent or progressive cancer, and APACHE II score are independent predictors of 30-day and 90-day mortality [3]. On the other hand, contrary to these studies, it was found in one study that the APACHE II severity score did not significantly predict ICU mortality when applied in patients with advanced lung cancer [23]. In our study, similar to the first two studies, an increase in the APACHE II score was found to be associated with increased mortality according to the results of multiple logistic regression analysis. It was determined to be the third-best test with regard to sensitivity and specificity, and the second-best test with respect to accuracy, PPV, NPV, and AUC.

Like APACHE II, the MPM II-Admission score is an important evaluation model used in patients and has been associated with OS. Groeger et al. tested the MPM II-Admission in a sample of 805 cancer patients and found that this model exhibited poor calibration and poor discrimination, and showed relatively lower mortality rates [24]. In another study, MPM II-Admission was similarly not identified as an independent predictor of poor prognosis with regard to 30-day mortality [25]. In our study, the MPM II-Admission scores of patients who died in ICU follow-up were found to be higher than those who were discharged. In addition, according to the results of the multivariable analysis, MPM II-Admission had the power to predict the probability of mortality independently from other tests. In addition to independent value in multiple logistic regression analysis, the MPM II-Admission score was found to yield the highest specificity, accuracy, PPV, NPV, and AUC values, while being the second-best test in terms of sensitivity. To provide a clearer comparative interpretation, ROC analyses were further conducted. Severity-based scores (MPM II-Admission and APACHE II) demonstrated markedly superior AUC, sensitivity, and specificity compared with inflammation-based indexes (NLR, PLR, LMR, SII, and mGPS). This indicates that physiologic derangement scores capture short-term mortality risk more effectively than systemic inflammation markers in the ICU oncology population. Among inflammation-based markers, only PNI showed clinically meaningful discriminatory ability, whereas NLR, PLR, LMR, and SII exhibited poor predictive value. To better contextualize discrimination performance, ROC analyses were evaluated comparatively. Severity-based scores (MPM II-Admission and APACHE II) demonstrated markedly superior AUC, sensitivity, and specificity compared with inflammation-based indexes (NLR, PLR, LMR, SII, and mGPS). This indicates that physiologic derangement scores capture short-term mortality risk more effectively than systemic inflammation markers in the ICU oncology population. Among inflammation-based markers, only PNI showed clinically meaningful discriminatory ability, whereas NLR, PLR, LMR, and SII exhibited poor predictive value.

Apart from these scores, we also found some self-explanatory results, such as that patients with recurrent or progressive lung cancer had a higher risk of death and that patients admitted to the ICU following surgery had a lower risk of death. It is already known that cancer recurrence and progression are associated with mortality, a finding which has been proven in many studies [26,27]. The results of our study are also in support of this knowledge.

NLR, PLR, and LMR have been the subjects of many studies [28,29,30,31]. For example, Diem et al. found that higher NLR and PLR were associated with lower overall survival in non-squamous cell lung cancer [1]. In another study, PLR and LMR were associated only with pre-treatment OS in lung cancer patients, whereas NLR was associated with both pre-treatment and post-treatment OS [2]. In a review that included a total of 20 studies involving 8304 patients, it was emphasized that patients with low LMR had lower overall survival compared to patients with high LMR and that pre-treatment LMR values could be used as an important prognostic marker in patients with non-squamous cell lung cancer [2]. Wang et al. studied 439 patients with stage I non-squamous cell lung cancer and demonstrated a positive correlation between LMR and overall survival, in addition to a greater risk of distal metastasis with lower LMR [29]. In another article investigating only NLR and PLR, it was shown that pre-treatment NLR and PLR values provided prognostic value in patients with non-squamous cell lung cancer and that when these two parameters were evaluated together, their predictive power increased [32]. In our study, NLR values of patients who died were found to be significantly lower than those who were discharged, but no such relationship was found for PLR and LMR in univariate analysis. Furthermore, the predictive values of PLR were very low for the identification of mortality development. More importantly, multivariable analyses showed that none of these simple, inexpensive parameters of inflammatory activity were independently associated with mortality. PNI integrates both nutritional reserves (albumin) and immunological competence (lymphocyte count), providing a multidimensional reflection of host vulnerability. This combined pathway may explain why PNI demonstrates stronger prognostic performance than inflammation-only markers (NLR, PLR, LMR, and SII) in critically ill lung cancer patients. Importantly, prior studies evaluating PNI in oncology populations have been conducted primarily in surgical cohorts or ambulatory patients. To our knowledge, the present study is the first to examine the prognostic significance of PNI specifically in an ICU-based lung cancer cohort, highlighting its unique relevance to acute critical illness.

The SII is another easily calculated and reliable parameter of inflammation that is obtained according to the values of three inflammatory cells (platelets, neutrophils, and lymphocytes) [33]. Existing studies show that SII is an independent prognostic factor in some malignant tumors, while there are studies showing that higher SII predicts worse survival in non-squamous cell lung cancer [34,35]. Strong associations were reported by Nøst et al. [20] and, in a meta-analysis, nine studies evaluating pre-treatment SII values in a total of 2441 patients showed that pre-treatment SII is a useful prognostic factor and also has a much higher prognostic value than both NLR and PLR [35]. Likewise, other researchers have also demonstrated that SII is a significant prognostic factor in patients with non-squamous cell lung cancer [33,34,36]. Contrary to previous studies, the performance of SII in predicting mortality is not significant in the current literature. In fact, it was one of the tests with the lowest sensitivity, specificity, accuracy, PPV, NPV, and AUC values.

Previous studies have suggested mGPS as a useful prognostic score for lung cancer [2,37]. This score is based on CRP and albumin, which reflect not only inflammatory status but also nutritional status [9,38]. In a review, data from 5817 patients gathered from 11 studies were evaluated. Pre-treatment mGPS data for patients with lung cancer and its correlation with OS values were evaluated and the results revealed that there was a significant relationship between high mGPS and overall survival in patients with lung cancer [9]. In the present study, mGPS was found to have the highest sensitivity but the lowest specificity. Although mGPS was higher in the exitus group compared to the discharged group, the mortality predictive power in multivariable logistic regression analysis was not statistically significant.

Recent evidence suggests that combining inflammatory markers (such as NLR, SII, and PNI) with ICU severity scores (e.g., APACHE II and MPM II) may provide a more accurate prognostic assessment than using single parameters alone. Multimarker approaches that integrate systemic inflammation, nutritional status, and physiologic derangement have been shown to increase discrimination power in several malignancy-related prognostic studies. This concept aligns with our findings: no single inflammatory marker demonstrated strong predictive value on its own, while PNI and severity-based scores showed complementary prognostic contributions. Incorporating composite models may therefore enhance early risk stratification in lung cancer patients admitted to the ICU.

Proinflammatory cytokines such as IL-6, IL-8, and sCD40L may offer additional mechanistic insights beyond inflammatory cell-derived indexes. Cytokine profiling could enhance prognostic assessment by capturing upstream inflammatory signaling. Future prospective studies incorporating cytokine measurements may therefore provide greater biological granularity in critically ill lung cancer populations. 

Future prospective studies should incorporate additional clinical endpoints, including organ dysfunction scores, vasopressor requirements, mechanical ventilation characteristics, duration of organ support, and functional outcomes. Incorporating these multidimensional clinical variables may provide a more comprehensive prognostic framework beyond survival alone. Future prospective multicenter studies with larger cohorts are required to perform net reclassification improvement (NRI) and decision-curve analyses to validate the clinical utility of these prognostic scores.

When taken together, our study shows that mGPS and PNI were the only inflammatory markers that had notable value in prognostic assessment of lung cancer patients admitted to the ICU. Although NLR demonstrated statistical significance in univariate and ROC analyses, low discriminatory capabilities prevented it from being identified as a marker that might be associated with the likelihood of death in this subset of patients. Most importantly, PNI was the only inflammation-related marker that was found to be independently associated with mortality. This information is important in terms of predicting mortality in the first admission to the intensive care unit. The identification of recurrence/progression status as a strong prognostic factor highlights the importance of early oncologic re-assessment and timely therapeutic interventions in ICU-admitted lung cancer patients. From a clinical standpoint, integrating PNI, APACHE II, and MPM II-Admission into routine risk stratification may facilitate rapid prognostic profiling at the time of ICU admission. These parameters are readily available, non-invasive, and practical for daily decision-making, allowing clinicians to refine goals of care, anticipate resource utilization, and guide communication with patients’ families. Future research should aim to validate these findings in multicenter cohorts and explore prognostic models that combine oncologic disease dynamics with systemic inflammation and severity-of-illness scores. Such models could enhance precision in prognostication and support more individualized management strategies in the ICU. Nonetheless, these conclusions must be assessed with respect to their pathophysiological relationships, which are needed to confirm our results.

## 5. Conclusions

In this study, we confirmed the prognostic value of PNI, APACHE II score, MPM II-Admission, and recurrence/progression status in lung cancer patients admitted to the ICU. Among all evaluated parameters, MPM II-Admission demonstrated the highest independent prognostic value for mortality. PNI, APACHE II, and MPM II-Admission appear to be practical, non-invasive, and easily accessible markers for prognostic assessment in this population. Notably, PNI was the only inflammation-related marker independently associated with mortality. Relevant limitations of our study should be acknowledged. This was a single-center study with a limited sample size, which may restrict the generalizability of the findings, particularly for inflammatory markers that show considerable interpatient variability. In addition, the retrospective design limits the strength of causal interpretations and highlights the need for prospective validation. Therefore, larger multicenter prospective studies are required to confirm our findings, explore the pathophysiological basis of these scores, and further define the clinical utility of inflammation-related markers in ICU-admitted lung cancer patients. PNI, APACHE II, and MPM II-Admission demonstrated promising prognostic performance in this single-center retrospective cohort; however, prospective multicenter validation is required before routine clinical adoption.

## 6. Limitations

This study has several limitations. The small number of SCLC patients prevented reliable subgroup analysis. Cytokine biomarkers (IL-6, IL-8, and sCD40L) could not be evaluated because they are not routinely measured in our ICU and no stored serum samples were available. The retrospective design limited assessment of key confounders such as cumulative chemotherapy exposure, timing of last treatment, and detailed nutritional status; however, supplementary analyses using available variables showed no meaningful effects on the PNI–mortality association. Mechanical ventilation and vasopressor use were excluded from multivariable models due to strong collinearity with APACHE II and MPM II-Admission. Lastly, the single-center retrospective nature of the study limits generalizability, and prospective multicenter validation is required.

## Figures and Tables

**Table 1:** Summary of patient characteristics and laboratory measurements with regard to groups.

		Status		
	Total (n = 229)	Exitus (n = 135)	Discharged (n = 94)	*p*
Age	66.17 ± 11.89	66.80 ± 12.20	65.27 ± 11.44	0.338
Sex				
Male	163 (71.18%)	94 (69.63%)	69 (73.40%)	0.535
Female	66 (28.82%)	41 (30.37%)	25 (26.60%)	
Diagnosis				
Small cell lung cancer	46 (20.09%)	30 (22.22%)	16 (17.02%)	0.424
Non-small cell lung cancer	183 (79.91%)	105 (77.78%)	78 (82.98%)	
Comorbidities	127 (55.46%)	69 (51.11%)	58 (61.70%)	0.113
Diabetes mellitus	42 (18.34%)	22 (16.30%)	20 (21.28%)	0.433
Hypertension	54 (23.58%)	31 (22.96%)	23 (24.47%)	0.916
Ischemic heart diseases	29 (12.66%)	14 (10.37%)	15 (15.96%)	0.294
COPD	39 (17.03%)	18 (13.33%)	21 (22.34%)	0.108
Hypothyroidism	5 (2.18%)	2 (1.48%)	3 (3.19%)	0.403
Atrial fibrillation	32 (13.97%)	16 (11.85%)	16 (17.02%)	0.360
Chronic renal failure	8 (3.49%)	5 (3.70%)	3 (3.19%)	1.000
Congestive heart failure	9 (3.93%)	4 (2.96%)	5 (5.32%)	0.493
Others	4 (1.75%)	2 (1.48%)	2 (2.13%)	1.000
Chemotherapy				
None	36 (16.90%)	15 (11.81%)	21 (24.42%)	**0.035**
<2 weeks	50 (23.47%)	29 (22.83%)	21 (24.42%)	
2–4 weeks	33 (15.49%)	25 (19.69%)	8 (9.30%)	
>4 weeks	94 (44.13%)	58 (45.67%)	36 (41.86%)	
Malignancy status				
Controlled/Remission	30 (15.08%)	12 (10.00%)	18 (22.78%)	**<0.001**
Newly diagnosed	46 (23.12%)	20 (16.67%)	26 (32.91%)	
Recurrence/Progression	123 (61.81%)	88 (73.33%)	35 (44.30%)	
Stage				
Stage I	4 (1.97%)	1 (0.81%)	3 (3.75%)	0.089
Stage II	4 (1.97%)	1 (0.81%)	3 (3.75%)	
Stage III	8 (3.94%)	3 (2.44%)	5 (6.25%)	
Stage IV	187 (92.12%)	118 (95.93%)	69 (86.25%)	
Reason of ICU admission				
Respiratory problems	131 (57.21%)	86 (63.70%)	45 (47.87%)	**<0.001**
Neurological problems	21 (9.17%)	10 (7.41%)	11 (11.70%)	
Sepsis	28 (12.23%)	22 (16.30%)	6 (6.38%)	
Postoperative	29 (12.66%)	3 (2.22%)	26 (27.66%)	
GI bleeding	6 (2.62%)	5 (3.70%)	1 (1.06%)	
Cardiac problems	5 (2.18%)	2 (1.48%)	3 (3.19%)	
Others	9 (3.93%)	7 (5.19%)	2 (2.13%)	
Length of stay in ICU	5 (2–9)	5 (2–11)	4 (2–8)	0.307
MV	196 (85.59%)	131 (97.04%)	65 (69.15%)	**<0.001**
Invasive MV	136 (59.39%)	105 (77.78%)	31 (32.98%)	**<0.001**
Hemoglobin (g/dL)	10.54 ± 2.05	10.07 ± 1.92	11.21 ± 2.05	**<0.001**
Platelet (×1000)	191 (104–302)	164 (69–293)	220 (150–312)	**0.007**
WBC (×10^3^/µL)	11,100 (6920–17,230)	11,480 (5670–18,830)	10,970 (7220–15,550)	0.656
Neutrophil	9410 (5350–14,900)	9840 (5050–15,690)	8680 (5510–13,430)	0.332
Lymphocyte	730 (340–1230)	620 (310–1100)	810 (340–1550)	**0.017**
Eosinophil	10 (0–30)	0 (0–20)	10 (0–60)	**0.002**
Monocyte	560 (230–980)	520 (190–890)	585 (270–1000)	0.353
CRP (mg/L)	120.6 (49.6–235.8)	140.07 (61.74–284.58)	109.63 (33.11–166.48)	**0.001**
Albumin (g/dL)	2.91 ± 0.59	2.82 ± 0.57	3.05 ± 0.60	**0.004**
LDH (U/L)	405 (246–787)	521 (311–1338)	284.5 (212–443)	**0.009**
NLR	12.38 (5.82–26.2)	14.92 (6.79–28.00)	9.85 (5.40–20.98)	**0.016**
PLR	272.5 (125.6–537.9)	267.19 (124.03–548.39)	280.71 (142.05–504)	0.808
LMR	1.37 (0.77–2.33)	1.25 (0.64–2.50)	1.46 (0.90–2.16)	0.334
SII (×1000)	2166 (1017.5–5094.7)	2128.3 (932.1–5703.1)	2183.9 (1088.1–4430.2)	0.907
mGPS				
0	12 (5.45%)	1 (0.74%)	11 (12.94%)	**<0.001**
1	36 (16.36%)	19 (14.07%)	17 (20.00%)	
2	172 (78.18%)	115 (85.19%)	57 (67.06%)	
PNI	33.55 ± 7.84	32.33 ± 7.18	35.54 ± 8.50	**0.004**
APACHE score	21.80 ± 8.84	25.03 ± 8.28	17.17 ± 7.49	**<0.001**
MPM II-Admission	58 (43–80)	71 (56–90)	44.5 (30–55)	**<0.001**

Data are given as mean ± standard deviation or median (1st quartile–3rd quartile) for continuous variables according to normality of distribution and as frequency (percentage) for categorical variables. COPD: Chronic obstructive pulmonary disease, ICU: intensive care unit, MV: mechanical ventilation, WBC: white blood cell, CRP: C-reactive protein, LDH: lactate dehydrogenase, NLR: neutrophil lymphocyte ratio, PLR: platelet lymphocyte ratio, LMR: lymphocyte monocyte ratio, SII: systemic immune-inflammation index, mGPS: modified Glasgow prognostic score, PNI: prognostic nutritional index. Bold values indicate *p* < 0.05.

**Table 2:** Performance of prognostic scores to predict mortality.

	Cut-off	Sensitivity	Specificity	Accuracy	PPV	NPV	AUC (95.0% CI)	*p*
NLR	≥12.5	59.40%	63.83%	61.23%	69.91%	52.63%	0.594 (0.519–0.670)	0.016
PLR	≥200	60.90%	43.62%	53.74%	60.45%	44.09%	0.491 (0.415–0.566)	0.808
LMR	<1.01	41.35%	68.09%	52.42%	64.71%	45.07%	0.538 (0.463–0.613)	0.334
SII	≥3150	39.85%	65.96%	50.66%	62.35%	43.66%	0.505 (0.429–0.580)	0.907
mGPS	2	85.19%	32.94%	65.00%	66.86%	58.33%	0.599 (0.520–0.678)	0.013
PNI	<31.1	48.85%	73.75%	58.29%	75.29%	46.83%	0.611 (0.533–0.689)	0.007
APACHE score	≥20	70.37%	69.15%	69.87%	76.61%	61.90%	0.762 (0.699–0.824)	<0.001
MPM II-Admission	≥59	71.85%	84.04%	76.86%	86.61%	67.52%	0.829 (0.775–0.884)	<0.001

PPV: positive predictive value, NPV: negative predictive value, AUC: area under ROC curve, CI: confidence intervals, NLR: neutrophil lymphocyte ratio, PLR: platelet lymphocyte ratio, LMR: lymphocyte monocyte ratio, SII: systemic immune-inflammation index, mGPS: modified Glasgow prognostic score, PNI: prognostic nutritional index. Bold values indicate *p* < 0.05.

**Table 3:** Significant prognostic factors of mortality, multiple logistic regression analysis.

	β Coefficient	Standard Error	*p*	Exp(β)	95.0% CI for Exp(β)	
Recurrence/Progression	1.880	0.503	<0.001	6.553	2.446	17.553
Postoperative admission	−3.452	1.095	0.002	0.032	0.004	0.271
PNI (<31.1)	0.940	0.472	0.047	2.559	1.014	6.455
APACHE score (≥20)	1.051	0.458	0.022	2.860	1.165	7.024
MPM II-Admission (≥59)	3.376	0.623	<0.001	29.257	8.635	99.128
(Constant)	−2.360	0.540	<0.001	0.094		
Dependent Variable: Mortality; Nagelkerke R^2^ = 0.632; Correct prediction = 85.87%						

CI: confidence interval, PNI: prognostic nutritional index.

## Data Availability

The original contributions presented in this study are included in the article. Further inquiries can be directed to the corresponding author.
